# Supplementary material for: Identification and Roles of miR-29b-1-3p and miR29a-3p-Regulated and Non-Regulated lncRNAs in Endocrine-Sensitive and Resistant Breast Cancer Cells
Source: Cancers (Basel). 2021 Jul 14;13(14):3530. doi: 10.3390/cancers13143530 (PMC8307416; doi:10.3390/cancers13143530)
Supplement: Supplementary file 1 [file cancers-13-03530-s001.zip › cancers-1267875-supplementary.pdf]

Supplementary Tables

Table S1: GO Processes for miR-29b-1-3p/miR-29a-3p- down-regulated lncRNAs in MCF-7 and LCC9 cells from Table 1 were identified by MetaCore.

Enrichment by GO Processes					lncRNA MCF-7 AS-miR29a (Table 1)				lncRNA LCC9 AS-miR29a (Table 1)			
#	**Processes**	**Total**	**Min (pValue)**	**Min FDR**	**p-value**	**FDR**	**In Data**	**Network Objects from Active Data**	**p-value**	**FDR**	**In Data**	**Network Objects from Active Data**
1	photoreceptor cell development	93	1.485E-02	1.442E-01	1.485E-02	1.442E-01	1	*TUG1*	1.485E-02	1.442E-01	1	*TUG1*
2	photoreceptor cell differentiation	113	1.803E-02	1.442E-01	1.803E-02	1.442E-01	1	*TUG1*	1.803E-02	1.442E-01	1	*TUG1*
3	neuron development	1360	2.012E-01	8.215E-01	2.012E-01	8.215E-01	1	*TUG1*	2.012E-01	8.215E-01	1	*TUG1*
4	neuron differentiation	1659	2.410E-01	8.215E-01	2.410E-01	8.215E-01	1	*TUG1*	2.410E-01	8.215E-01	1	*TUG1*
5	generation of neurons	2025	2.877E-01	8.215E-01	2.877E-01	8.215E-01	1	*TUG1*	2.877E-01	8.215E-01	1	*TUG1*
6	neurogenesis	2210	3.105E-01	8.215E-01	3.105E-01	8.215E-01	1	*TUG1*	3.105E-01	8.215E-01	1	*TUG1*
7	cell development	2624	3.594E-01	8.215E-01	3.594E-01	8.215E-01	1	*TUG1*	3.594E-01	8.215E-01	1	*TUG1*
8	nervous system development	3383	4.424E-01	8.847E-01	4.424E-01	8.847E-01	1	*TUG1*	4.424E-01	8.847E-01	1	*TUG1*
9	cell differentiation	5330	6.184E-01	9.315E-01	6.184E-01	9.315E-01	1	*TUG1*	6.184E-01	9.315E-01	1	*TUG1*
10	cellular developmental process	5397	6.236E-01	9.315E-01	6.236E-01	9.315E-01	1	*TUG1*	6.236E-01	9.315E-01	1	*TUG1*

Table S2: Networks and GO Processes identified for miR-29b-1-3p/miR-29a-3p-down-regulated lncRNAs in MCF-7 and LCC9 cells from Table 1 were identified by MetaCore.

	Network	GO Processes	Total nodes	Seed nodes	p-Value	zScore
1.	*JPX, miR-33a-3p*		2	1	1.220E-04	90.41
2.	*TUG1, CRNDE, APTR, DANCR, SNHG5*	system development (84.1%; 4.224e-15), cellular response to hormone stimulus (43.2%; 7.037e-15), protein-containing complex subunit organization (54.5%; 1.842e-14), response to hormone (50.0%; 2.029e-14), response to organic cyclic compound (52.3%; 3.072e-14)	50	5	1.020E-15	90.37
3.	*DLEU7-AS1, Gas5, SOX2OT, LINC00152, TUG1*	chromatin disassembly (19.0%; 1.072e-16), nucleosome disassembly (19.0%; 1.072e-16), protein-DNA complex disassembly (19.0%; 5.049e-16), positive regulation of macromolecule biosynthetic process (61.9%; 3.763e-15), positive regulation of cellular metabolic process (76.2%; 4.024e-15)	50	5	1.020E-15	90.37
4.	*DLEU1, MIR99AHG, FOXP3, KCNMB4,* nAChR delta *(CHRND)*	excitatory postsynaptic potential (28.6%; 3.305e-22), chemical synaptic transmission, postsynaptic (28.6%; 4.190e-21), regulation of postsynaptic membrane potential (28.6%; 1.682e-18), chemical synaptic transmission (34.7%; 8.585e-15), anterograde trans-synaptic signaling (34.7%; 8.585e-15)	50	2	3.830E-06	37.25
5.	*GAS5, CREB1, LAMC2,* PKA-cat alpha, Stearic acid + ATP + CoA = Octadecanoyl-CoA + AMP + Pyrophosphate	cell development (57.5%; 8.446e-13), plasma membrane bounded cell projection organization (47.5%; 3.089e-12), neurogenesis (52.5%; 3.601e-12), cell projection organization (47.5%; 5.857e-12), cellular component organization (82.5%; 1.754e-11)	50	1	2.810E-03	18.8

6.	*SNHG5*, *FOXP3*, Thioredoxin, *SPATS2*, ADP + Thioredoxin = H(,2)O + Thioredoxin oxidized + dADP	stimulatory C-type lectin receptor signaling pathway (15.9%; 9.953e-09), innate immune response activating cell surface receptor signaling pathway (15.9%; 1.374e-08), innate immune response-activating signal transduction (15.9%; 1.571e-08), SCF complex assembly (9.1%; 3.154e-08), mitotic cell cycle process (27.3%; 3.655e-08)	50	1	2.870E-03	18.6
7.	*MIR17HG*, Beta-catenin (*CTNNB1*), *FAM124B*, *MRPS24*, nAChR alpha-3 (*CHRNA3*)	cellular amide metabolic process (31.9%; 8.401e-10), organonitrogen compound biosynthetic process (38.3%; 2.654e-09), amide biosynthetic process (25.5%; 5.495e-09), fatty-acyl-CoA biosynthetic process (10.6%; 5.726e-09), fatty-acyl-CoA metabolic process (10.6%; 2.895e-08)	50	1	2.990E-03	18.21

Table S3: GO Processes for miR-29b-1-3p/miR-29a-3p- down-regulated lncRNAs in MCF-7 and LCC9 cells from Table 1 were identified by MetaCore.

**Enrichment by GO Processes**					**lncRNA MCF-7 AS-miR29a (Table 10**				**lncRNA LCC9 AS-miR29a (Table 1)**			
**#**	**Processes**	**Total**	**Min (pValue)**	**Min FDR**	**p-value**	**FDR**	**In Data**	**Network Objects from Active Data**	**p-value**	**FDR**	**In Data**	**Network Objects from Active Data**
1	nuclear speck organization	5	4.015E-04	1.124E-02	4.015E-04	1.124E-02	1	*MALAT1*	4.015E-04	1.124E-02	1	*MALAT1*
2	nuclear body organization	21	1.686E-03	2.360E-02	1.686E-03	2.360E-02	1	*MALAT1*	1.686E-03	2.360E-02	1	*MALAT1*
3	regulation of alternative mRNA splicing, via spliceosome	91	7.295E-03	5.722E-02	7.295E-03	5.722E-02	1	*MALAT1*	7.295E-03	5.722E-02	1	*MALAT1*
4	cellular component maintenance	102	8.175E-03	5.722E-02	8.175E-03	5.722E-02	1	*NEAT1*	8.175E-03	5.722E-02	1	*NEAT1*
5	regulation of mRNA splicing, via spliceosome	146	1.169E-02	5.739E-02	1.169E-02	5.739E-02	1	*MALAT1*	1.169E-02	5.739E-02	1	*MALAT1*
6	nucleus organization	184	1.472E-02	5.739E-02	1.472E-02	5.739E-02	1	*MALAT1*	1.472E-02	5.739E-02	1	*MALAT1*
7	regulation of mRNA processing	195	1.560E-02	5.739E-02	1.560E-02	5.739E-02	1	*MALAT1*	1.560E-02	5.739E-02	1	*MALAT1*
8	regulation of RNA splicing	205	1.640E-02	5.739E-02	1.640E-02	5.739E-02	1	*MALAT1*	1.640E-02	5.739E-02	1	*MALAT1*
9	regulation of synapse organization	360	2.870E-02	8.436E-02	2.870E-02	8.436E-02	1	*MALAT1*	2.870E-02	8.436E-02	1	*MALAT1*
10	regulation of synapse structure or activity											

Table S4: Networks and GO Processes identified for miR-29b-1-3p/miR-29a-3p-down-regulated lncRNAs in MCF-7 and LCC9 cells from Table 1 were identified by MetaCore.

	Network	GO Processes	Total nodes	Seed nodes	p-Value	zScore
1.	*NEAT1*, *UCA1*, *MALAT1*, *TINCR*, *SMAD2*	histone monoubiquitination (13.2%), negative regulation of biological process (76.3%), histone ubiquitination (13.2%), positive regulation of protein catabolic process (21.1%), histone H2A monoubiquitination (10.5%)	50	4	5.79e- 14	108.37
2.	*ABHD11 AS1*, miR 1254, *RBBP6*	somite development (100.0%), regulation of DNA replication (100.0%), multicellular organism growth (100.0%), DNA replication (100.0%), protein polyubiquitination (100.0%)	3	1	8.69e- 05	107.25
3.	*NEAT1*, *MALAT1*, *UCA1*, miR 185 5p, *SMAD1*	positive regulation of epithelial to mesenchymal transition (20.8%), gastrulation (29.2%), regulation of epithelial cell differentiation involved in kidney development (16.7%), embryonic morphogenesis (37.5%), regulation of epithelial to mesenchymal transition (20.8%)	31	3	7.56e- 11	100.08

Table S5: lncRNAs differentially expressed in MCF-7 and LCC9 cells that were not regulated by miR-29b-1-3p/miR-29a-3p-with low expression < 1 FPKM. Values are FPKM and are the average of 15 biological replicates +/- Standard deviation.

	Ensembl	Gene name	Alias	MCF-7 avg	sem	LCC9 avg	sem	comparison	References in breast cancer
1.	ENSG00000253438	*PCAT1*		0.14	0.05	0.24	0.02	LCC9 > MCF-7	Elevated, binds RACK1 to prevent degradation of HIF1-α [275]
2.	ENSG00000246228	*CASC8*	LINC00860	0.16	0.04	0.99	0.10	LCC9 > MCF-7	A meta-analysis correlating CASC8 to breast cancer [276]
3.	ENSG00000197291	*RAMP2-AS1*	CTD-3193K9.7	0.24	0.05	0.42	0.02	LCC9 > MCF-7	
4.	ENSG00000245750	*DRAIC*	RP11-279F6.1 (lncRNA152)	0.27	0.08	2.43	0.40	LCC9 > MCF-7	Regulates estrogen signaling in breast cancer [277].<br><br>Expression is negatively correlated with immune cell infiltration [278].
5.	ENSG00000249375	*CASC11*	RP11-1136L8.1	0.36	0.01	0.23	0.05	MCF-7 > LCC9	
6.	ENSG00000270419	*CAHM*	LINC00468	0.34	0.03	0.10	0.02	MCF-7 > LCC9	Low expression associated with low OS in TNBC [279]
7.	ENSG00000253552	*HOXA-AS2*		0.40	0.02	0.18	0.04	MCF-7 > LCC9	High expression associated with distant metastasis and sponges miR-520-3p to increase TGFBR2 and REL in BC cell lines [280]
8.	ENSG00000254349	*MIR2052HG*	RP11-758M4.1	0.34	0.05	2.11	0.17	LCC9 > MCF-7	Sustains ERα by interacting with EGR1 to facilitate its recruitment to the LMTK2 promoter, reducing PKC which increases AKT/FOXO3-mediated ESR1 transcription and by limiting ubiquitin-mediated, proteasome-dependent degradation [281,282]
9.	ENSG00000245573	*BDNF-AS*		0.44	0.04	0.19	0.05	MCF-7 > LCC9	Lower in breast tumors than normal breast [283]. In contrast, another report shows higher BDNF-AS in TNBC and endocrine resistant breast tumors compared to endocrine sensitive tumors [284]. Increased in TAM-R MCF-7, TAM-R T47D relative to parental MCF-7 and T47D cells [284]. Binds

									RNH1 and recruits TRIM21 for ubiquitinylation and degradation of RNH1 thus activating mTOR [284]
10.	ENSG00000272168	*CASC15*	LINC00340	0.40	0.06	0.15	0.04	MCF-7 > LCC9	Upregulated in breast cancer and functions as an oncogene by sponging miR-153-3p and upregulating KLF5 (reviewed in [285]).
11.	ENSG00000253929	*CASC21*	RP11-382A18.2	0.45	0.08	1.13	0.13	LCC9 > MCF-7	
12.	ENSG00000258325	*ITFG2-AS1*	RP4-816N1.6	0.68	0.14	1.09	0.20	LCC9 > MCF-7	
13.	ENSG00000232044	*SILC1*	AC073479.1, LINC01105	0.89	0.23	0.00	0.00	MCF-7 > LCC9	
14.	ENSG00000250786	*SNHG18*	CTD-2001E22.2	0.89	0.15	0.00	0.00	MCF-7 > LCC9	
15.	ENSG00000240990	*HOXA11-AS*	HOTAIR	0.70	0.11	0.22	0.06	MCF-7 > LCC9	Upregulated in breast tumors and cell lines and knockdown reduced MDA-MB-231 cell proliferation, invasion, and migration *in vitro* and tumor xenograft growth and tail vein injected 'lung metastasis' in vivo [286].
16.	ENSG00000265962	*GACAT2*	RP11-674N23.1	0.62	0.18	0.01	0.00	MCF-7 > LCC9	
17.	ENSG00000228126	*FALEC*	LINC00568	0.85	0.09	0.49	0.08	MCF-7 > LCC9	

Table S6: GO Processes for lncRNAs differentially expressed in MCF-7 and LCC9 cells from Table 3 were identified by MetaCore.

Enrichment by GO Processes					lncRNA MCF7 ASmiR29aTable1				lncRNA LCC9 ASmiR29aTable1			
**#**	**Processes**	**Total**	**Min (pValue)**	**Min FDR**	**p-value**	**FDR**	**In Data**	**Network Objects from Active Data**	**p-value**	**FDR**	**In Data**	**Network Objects from Active Data**
1	mesenchymal to epithelial transition	23	3.689E-03	1.199E-01	3.689E-03	1.199E-01	1	*H19*	3.689E-03	1.199E-01	1	*H19*
2	dosage compensation by inactivation of X chromosome	31	4.970E-03	1.199E-01	4.970E-03	1.199E-01	1	*XIST*	4.970E-03	1.199E-01	1	*XIST*
3	dosage compensation	33	5.290E-03	1.199E-01	5.290E-03	1.199E-01	1	*XIST*	5.290E-03	1.199E-01	1	*XIST*
4	gene silencing by miRNA	78	1.247E-02	1.774E-01	1.247E-02	1.774E-01	1	C17orf91	1.247E-02	1.774E-01	1	C17orf91
5	post-transcriptional gene silencing by RNA	90	1.438E-02	1.774E-01	1.438E-02	1.774E-01	1	C17orf91	1.438E-02	1.774E-01	1	C17orf91
6	posttranscriptional gene silencing	98	1.565E-02	1.774E-01	1.565E-02	1.774E-01	1	C17orf91	1.565E-02	1.774E-01	1	C17orf91
7	gene silencing by RNA	131	2.088E-02	2.028E-01	2.088E-02	2.028E-01	1	C17orf91	2.088E-02	2.028E-01	1	C17orf91
8	skeletal muscle tissue development	242	3.831E-02	2.796E-01	3.831E-02	2.796E-01	1	*H19*	3.831E-02	2.796E-01	1	*H19*
9	skeletal muscle organ development	260	4.111E-02	2.796E-01	4.111E-02	2.796E-01	1	*H19*	4.111E-02	2.796E-01	1	*H19*
10	gene silencing	260	4.111E-02	2.796E-01	4.111E-02	2.796E-01	1	C17orf91	4.111E-02	2.796E-01	1	C17orf91

Table S7: Networks and GO Processes identified for miR-29b-1-3p/miR-29a-3p- down-regulated lncRNAs in MCF-7 and LCC9 cells from Table 1 were identified by MetaCore.

	Network	GO Processes	Total nodes	Seed nodes	p-Value	zScore
1.	LOC647979, NBR2, PCGEM1, LINC-PINT, MIR600HG	membrane depolarization during cardiac muscle cell action potential (23.3%; 8.156e-21), membrane depolarization (27.9%; 4.843e-19), membrane depolarization during action potential (23.3%; 5.062e-19), cardiac conduction (27.9%; 5.598e-18), cardiac muscle cell contraction (23.3%; 2.554e-17)	50	7	3.490E-21	101.08
2.	PVT1, ZFAS1 RNA, XIST, SNHG1, DLEU2	positive regulation of nitrogen compound metabolic process (76.7%; 1.584e-16), positive regulation of macromolecule metabolic process (76.7%; 4.378e-15), positive regulation of cellular metabolic process (74.4%; 1.276e-14), regulation of gene expression (83.7%; 1.688e-14), positive regulation of metabolic process (76.7%; 4.342e-14)	50	5	1.490E-14	70.72
3.	PCGEM1, ZFAS1 RNA, ZEB1-AS1, HOTAIR, ESR1 (nuclear)	cell-cell adhesion (41.9%; 1.773e-16), T cell proliferation (23.3%; 3.267e-16), activated T cell proliferation (16.3%; 1.976e-15), cellular response to organic substance (69.8%; 2.800e-15), cell population proliferation (41.9%; 3.603e-15)	50	4	1.860E-11	56.56
4.	H19, XIST, HOTAIRM1, SNAIL1, Cyclin A2	transcription initiation from RNA polymerase II promoter (31.8%; 5.974e-18), DNA-templated transcription, initiation (31.8%; 1.307e-16), regulation of cell cycle (54.5%; 1.348e-16), response to endogenous stimulus (61.4%; 9.759e-16), response to organic cyclic compound (54.5%; 2.346e-15)	50	3	1.760E-08	42.4
5.	HOTAIRM1, FTX, c-Src, miR-320-3p, CACNA1C	vascular endothelial growth factor receptor signaling pathway (21.3%; 1.182e-14), negative regulation of macrophage derived foam cell differentiation (14.9%; 4.974e-14), cell surface receptor signaling pathway (59.6%; 3.727e-13), regulation of protein localization (42.6%; 5.049e-13), regulation of protein transport (36.2%; 8.433e-13)	50	2	1.180E-05	28.25

6.	ANRIL, HOTAIR, RARbeta, Syk, CDK4	positive regulation of nitrogen compound metabolic process (91.5%; 2.079e-27), positive regulation of cellular metabolic process (91.5%; 2.679e-26), positive regulation of macromolecule metabolic process (91.5%; 1.980e-25), positive regulation of metabolic process (91.5%; 4.714e-24), regulation of gene expression (95.7%; 2.090e-23)	50	2	1.180E-05	28.25
7.	PCGEM1, FASN, VDR, IL-6, Hexanoyl-ACP + Malonyl-ACP = Acyl-carrier protein + HCO(,3)('-) + 3-Oxooctanoyl-ACP	positive regulation of transcription, DNA-templated (88.9%; 5.214e-29), positive regulation of nucleic acid-templated transcription (88.9%; 1.624e-28), positive regulation of RNA biosynthetic process (88.9%; 1.646e-28), positive regulation of transcription by RNA polymerase II (83.3%; 4.264e-28), positive regulation of RNA metabolic process (88.9%; 7.710e-28)	50	1	3.690E-03	16.4
8.	FTX, Beta-catenin, PANK2, DAAM2, PPTC7	multicellular organismal signaling (54.2%; 1.324e-41), calcium ion transmembrane transport (56.2%; 7.753e-41), cardiac conduction (47.9%; 3.225e-40), calcium ion transport (56.2%; 5.167e-37), regulation of heart contraction (56.2%; 1.106e-36)	50	1	4.780E-03	14.38
9.	DLEU2, E2F1, Fe('2+) + H('+) + O(,2) = Fe('3+) + H(,2)O, PtdIns(3,4,5)P3 intracellular, GSK3 alpha/beta	negative regulation of biological process (78.6%; 4.016e-10), cellular macromolecule catabolic process (35.7%; 4.188e-10), regulation of cell cycle (40.5%; 1.527e-09), cellular macromolecule metabolic process (71.4%; 2.697e-09), regulation of metabolic process (83.3%; 2.741e-09)	50	1	4.780E-03	14.38
10.	NBR2, Beta-catenin, PP2C alpha, SH2B, MED12	response to organic cyclic compound (51.1%; 1.687e-14), blood coagulation (29.8%; 5.686e-14), coagulation (29.8%; 6.263e-14), hemostasis (29.8%; 6.892e-14), wound healing (34.0%; 1.160e-13)	50	1	4.880E-03	14.23
